# Cell-type specific gene expression profiles of leukocytes in human peripheral blood

**DOI:** 10.1186/1471-2164-7-115

**Published:** 2006-05-16

**Authors:** Chana Palmer, Maximilian Diehn, Ash A Alizadeh, Patrick O Brown

**Affiliations:** 1 Department of Genetics, Stanford University School of Medicine, Stanford, USA; 2 Department of Biochemistry, Stanford University School of Medicine, Stanford, USA; 3 Department of Radiation Oncology, Stanford University School of Medicine, Stanford, USA; 4 Department of Hematology, Stanford University School of Medicine, Stanford, USA; 5 Howard Hughes Medical Institute, Stanford University School of Medicine, Stanford, USA

## Abstract

**Background:**

Blood is a complex tissue comprising numerous cell types with distinct functions and corresponding gene expression profiles. We attempted to define the cell type specific gene expression patterns for the major constituent cells of blood, including B-cells, CD4+ T-cells, CD8+ T-cells, lymphocytes and granulocytes. We did this by comparing the global gene expression profiles of purified B-cells, CD4+ T-cells, CD8+ T-cells, granulocytes, and lymphocytes using cDNA microarrays.

**Results:**

Unsupervised clustering analysis showed that similar cell populations from different donors share common gene expression profiles. Supervised analyses identified gene expression signatures for B-cells (427 genes), T-cells (222 genes), CD8+ T-cells (23 genes), granulocytes (411 genes), and lymphocytes (67 genes). No statistically significant gene expression signature was identified for CD4+ cells. Genes encoding cell surface proteins were disproportionately represented among the genes that distinguished among the lymphocyte subpopulations. Lymphocytes were distinguishable from granulocytes based on their higher levels of expression of genes encoding ribosomal proteins, while granulocytes exhibited characteristic expression of various cell surface and inflammatory proteins.

**Conclusion:**

The genes comprising the cell-type specific signatures encompassed many of the genes already known to be involved in cell-type specific processes, and provided clues that may prove useful in discovering the functions of many still unannotated genes. The most prominent feature of the cell type signature genes was the enrichment of genes encoding cell surface proteins, perhaps reflecting the importance of specialized systems for sensing the environment to the physiology of resting leukocytes.

## Background

Circulating leukocytes are a rich and readily accessible source of information about the health and physiological state of an individual. A procedure as simple as light microscopy-based quantitation of morphologically distinguishable blood cell types is so broadly useful that it has been a mainstay of clinical diagnosis for decades. Methods that might resolve more subtle variations in leukocytes could have correspondingly greater diagnostic power [1]. To explore and develop this potential, gene expression profiling of peripheral blood cells has become an increasingly popular means of addressing a wide variety of questions about health and disease. This approach has been used to study numerous states of health including multiple sclerosis, renal cell carcinoma, stroke, smallpox, neurofibromatosis type 1, and responses to various stresses [2-8] in the hopes of developing easily assayable prognostic or diagnostic markers, and gaining insight into disease mechanisms, as well as to the study of natural variation and individuality in gene expression [9-11]. While many of these studies have been successful in identifying gene expression patterns that differentiate control and disease groups, their interpretation is often confounded by variation in relative proportions of the cell populations that make up whole blood.

Blood is a complex tissue, containing a variety of cell types – including T-cells, B-cells, monocytes, NK cells, and granulocytes, each of which can be further subdivided. The relative proportion of each of these cell types can vary greatly between individuals and with states of health and disease, and in response to stimuli. In whole blood, neutrophils are usually the most abundant cell type, normally varying in abundance from 30–70% of white blood cells in healthy adults [12,13] and even more (or less) in disease. Neutrophils are often excluded from analyses of gene expression in human blood, but the remaining mixture of peripheral blood mononuclear cells (PBMC), can also vary greatly in its composition. In healthy adults, monocytes can vary from 2 to 10% of PBMCs [12], and within the lymphocyte subset, the relative proportion of T-lymphocytes and B-lymphocytes can range from 61–85% and 7–23% respectively [14]; furthermore, the ratio of CD4+ T-cells to CD8+ T-cells can vary from <1.0 to 2.0 [15]. The relative proportions of the contributing cell types inevitably affect the composite gene expression profiles of whole blood or unfractionated PBMCs. Variation in the relative proportions of distinct cell types provides valuable clinical information in its own right. The ability to distinguish the effects of variation in cellular "demographics" from the signatures of physiological responses, in global gene expression profiles of peripheral blood samples, would thus undoubtedly improve our ability to extract physiological and clinical insights from these signatures.

By comparing gene expression profiles of homogeneous cell populations, it is possible to identify genes with cell-type-specific gene expression patterns. These sets of genes can serve as "biomarkers" for estimating the abundance of specific cell types, and can provide insights into cellular functioning. By sorting peripheral blood from healthy donors based on cell surface markers, we obtained purified populations of CD4+ T-cells, CD8+ T-cells, and B-cells. We then compared the global gene expression profiles of these purified populations, PBMCs and whole blood samples, and attempted to identify cell-type-specific signatures for B-cells, T-cells, CD4+ T-cells, CD8+ T-cells, lymphocytes, and granulocytes.

## Results and discussion

### Overview

We used DNA microarrays containing 37,632 elements representing ~18,000 genes to characterize the global gene expression profiles for 9 B-cell, 6 CD8+ T-cell, 5 CD4+ T-cell, 5 PBMC and 3 whole blood samples, representing 3 female and 4 male healthy adults. In order to obtain an overview of the major sources of variation in this data set, we selected a set of variably expressed genes (741 genes/1109 clones whose transcripts levels varied 3.0 fold or more from the mean across all samples, in at least 3 of the 28 samples) and clustered the samples and genes using these genes.

**Figure 1:**
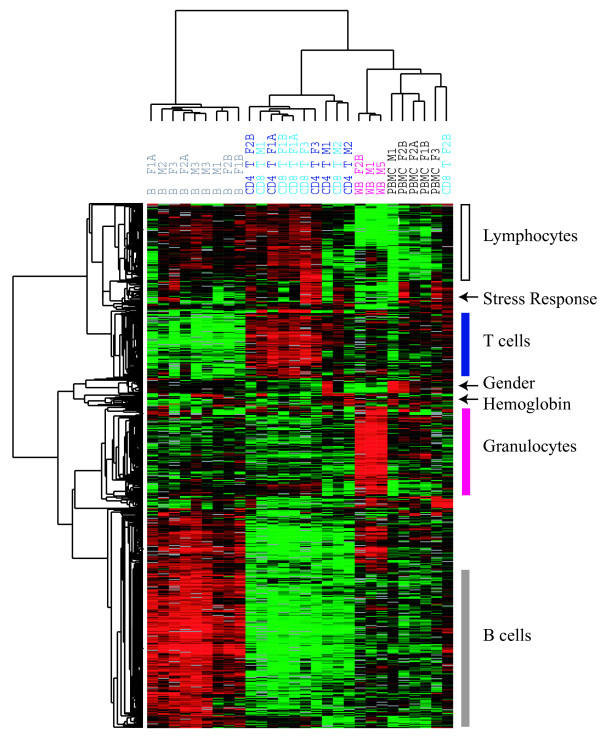
**Overview of hierarchical clustering of all samples**. Hierarchically clustered gene expression profiles of 28 mixed and purified leukocyte samples. Results for ~741 genes with high variation in transcript levels among these samples. Genes and blood samples are organized by hierarchical clustering based on overall similarity in expression patterns. Expression levels are represented by a color key in which bright red represents the highest levels and bright green represents the lowest levels, and less saturated shades represent intermediate levels of expression. Values were centered across all samples to a mean of zero. Clusters of genes are labeled according to which samples have the highest relative gene expression.

As shown in Figure 1, hierarchical clustering separated the samples into four main groups: B-cells, T-cells, whole blood, and PBMCs; notably, the two purified T-cell subsets (CD4+ and CD8+ T-cells) were intermingled. This analysis revealed sets of genes whose transcripts are characteristically expressed by specific cell types. The set of genes preferentially expressed in B-cells included many genes encoding immunoglobulins and proteins involved in MHC class II antigen processing and presentation, while the set of genes most highly expressed in T-cells was rich in genes encoding the T-cell receptor complex and associated signaling molecules. Both the B and T-cell enriched gene clusters also included cytokine receptors and cell adhesion receptors known to have cell-type-specific expression. The transcripts enriched in the whole blood samples appeared to reflect the transcriptional program of neutrophils, which are abundant in whole blood yet virtually absent from the lymphocyte and PBMC samples. This gene cluster included transcripts encoding several Fc receptors and granulocyte-specific cytokine receptors. The cluster of genes with the lowest relative expression in whole blood revealed genes more highly expressed in lymphoid than in myeloid cells. This cluster was almost entirely composed of ribosomal genes and other genes involved in translation. There were three distinct gene clusters that were not associated with a specific cell type. The first was a small gender-associated cluster, with the expected elevated expression of Y-linked genes in males, and of XIST in females. The second was a "stress response" associated cluster, which contained a set of genes known to be easily activated in response to ex-vivo handling and a variety of other stresses [16]. This set of genes was most highly activated in samples from female 3, all of which were obtained during the first (and longest) isolation experiment, and expressed at the lowest levels in the whole blood samples, which were subject to the least manipulation. A final small but prominent cluster was the hemoglobins – alpha, beta, and zeta. These were highly expressed in the whole blood samples and in a subset of the B-cell samples, corresponding to those B-cell samples that were purified by negative selection (depletion of non-B-cells) rather than positive selection and presumably represented the relative proportion of RBCs in these samples. There were no other consistent differences between the positively and negatively selected B-cells, although the latter were less pure than the positively selected B-cells and had noticeably more red blood cell contamination. All T-cell samples were obtained by negative selection in order to minimize cell stimulation [17]. See Additional file 1 for the complete data set.

For each cell population of interest (B-cells, T-cells, CD4+ T-cells, CD8+ T-cells, granulocytes and lymphocytes), we also performed a supervised search for genes that are characteristically expressed by that cell type. Specifically, for each cell type, we searched for genes whose expression was significantly positively correlated with the estimated relative abundance of the cell type across the set of 28 samples of varying cell type composition (p < 0.005 by permutation). We refined these gene lists by also requiring that a signature gene discriminate between the target cell and a closely related cell population (T-cells versus B-cells; CD4+ T-cells versus CD8+ T-cells; whole-blood versus PBMC), using a two-class Significance Analysis of Microarrays (SAM) [18] (False Discovery Rate < = 5%). We analyzed the final cell type specific gene lists for functional and structural themes using EASE [19], a program for calculating the statistical enrichment of Gene Ontology (GO) [20] annotations in a query list relative to a background list (Bonferroni p < 0.05 for Fisher's Exact Test).

### B-lymphocytes

**Figure 2:**
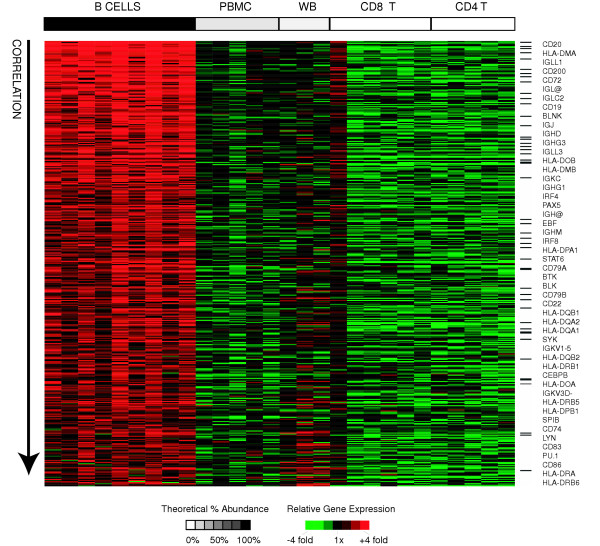
**B-cell signature genes**. Relative expression of B-cell signature genes is shown for all samples. Genes (rows) are sorted in descending order of correlation of gene expression with relative abundance of B-cells. Samples (columns) are ordered in decreasing order of estimated relative abundance of B-cells from left to right. The grayscale bar on top shows the estimated relative abundance of B-cells in each class of samples – black indicates 100% relative abundance of B-cells, white indicates 0% relative abundance of B-cells, and grey indicates intermediate relative abundance of B-cells. Gene expression values are centered across all samples to a median of zero. All genes mentioned in the text are listed in order of correlation with B-cell abundance and black bars indicate their positions in the figure.

B-lymphocytes function primarily in humoral (antibody-mediated) immunity, and comprise approximately 15% of lymphocytes in healthy adults. We derived a B-cell signature by searching for genes that met the following criteria: I) their expression was significantly positively correlated with relative abundance of B-cells across all samples II) their expression was significantly different in B-cells than T-cells (by SAM) and III) they were at least 2-fold more highly expressed in B-cells than in T-cells. Using these criteria, we identified 427 "B-cell signature" genes (represented by 814 clones) (Figure 2). This signature set included numerous genes encoding classical B-cell associated genes including B-cell co-receptor molecules (CD19, CD21, CD22, FCGR2B) [21], other B-cell surface markers (CD20, CD24, CD38, CD72, CD74, CD79A/B, CD83, CD86) [22], immunoglobulins (IG gamma, kappa, lambda; light and heavy), MHC class II receptors (HLA-DM/O/P/Q/R), signal transduction molecules (SYK, LYN, BTK, BLNK, BLK) and transcriptional regulators (EBF, PAX5/BSAP, OBF1/POU2AF1, SPIB, PU.1/SPI1, IRF4, IRF8, CEBPB) [23]. We analyzed the B-cell signature with EASE, and confirmed that the most enriched GO annotations were those relating to classical B-cell functions such as "antigen binding", "antigen processing", "antigen presentation", and "MHC Class II receptor activity". The only significantly enriched GO annotations relating to cellular component were "integral to membrane" and "membrane", which is consistent with the importance of interactions with the external environment in defining the unique characteristics of the B-cell. The complete list of B-cell specific genes, and results from EASE analysis are available as supplemental data (see Additional file 2 for gene list and Additional file 3 for EASE results).

### T-lymphocytes

**Figure 3:**
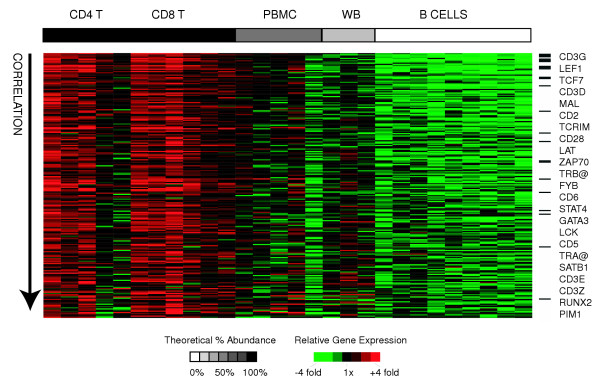
**T-cell signature genes**. Relative expression of T-cell signature genes is shown for all samples. Genes (rows) are sorted in descending order of correlation of gene expression with relative abundance of T-cells. Samples (columns) are ordered in decreasing relative abundance from left to right. Gene expression values are centered across all samples to a median of zero. The grayscale bar on top shows the estimated relative abundance of T-cells in each class of samples – black indicates 100% relative abundance of T-cells, white indicates 0% relative abundance of T-cells, and grey indicates intermediate relative abundance of T-cells. All genes mentioned in the text are listed in order of correlation with T-cell abundance, and black bars indicate their positions in the figure.

T-lymphocytes function primarily in cell-mediated immunity, and comprise approximately 70% of lymphocytes. We derived a T-cell signature by searching for genes that met the following criteria: I) their expression was significantly positively correlated with the relative abundance of T-cells (CD4+ or CD8+) across all samples II) their expression was significantly different between T-cells and B-cells (by SAM) and III) they were at least 2-fold more highly expressed in T-cells than in B-cells. Using these criteria, we identified 222 "T-cell signature" genes (represented by 370 clones) (Figure 3). This gene set encompassed many of the T-cell receptor and associated signal transduction genes (CD3δ/γ, CD28, TRα/β, MAL, LAT, TCRIM, FYN, ZAP70) [24] and also included genes encoding important T-cell transcriptional regulators (GATA3, LEF1, TCF7, RUNX2, STAT4, SATB1) [25] and cell adhesion molecules (CD2, CD5, CD6). The most enriched GO annotations (as determined with EASE) included the cellular component "T-cell receptor complex", the biological process "signal transduction", and the molecular function "receptor activity", suggesting that T-cells are best distinguished from other cell types based on their cell surface receptors and associated signal transduction genes.

We compared our list of 222 T-cell enriched genes with a recently published list of 92 "T-cell enriched" genes [26], and found that 33 of the 81 genes on this list that were also measured in our study, were also identified as T-cell enriched in this study (P < 10^-41^, hypergeometric distribution probability of > = 33 T-cell genes/81 genes given population ratio of 222 T-cell genes/16865 genes measured). The genes identified in common by the two studies tended to be classical T-cell associated genes with high discrimination rankings in both studies. This overlap is especially striking in view of the fact that the two studies used different microarray platforms (two color comparative cDNA hybridization versus one color Affymetrix oligonucleotide arrays) and, more importantly, the different selection criteria for T-cell specificity – we required discrimination of T-cells from B-cells as well as a significantly positive correlation between a gene's expression and T-cell abundance across all mixed and purified samples, whereas Cobb *et al*. required only discrimination between of T-cells and mixed blood cells. The complete list of T-cell specific genes and the results of EASE analysis are available as supplemental data (see Additional file 2 for gene list and Additional file 3 for EASE results).

### CD8+ T-cells

**Figure 4:**
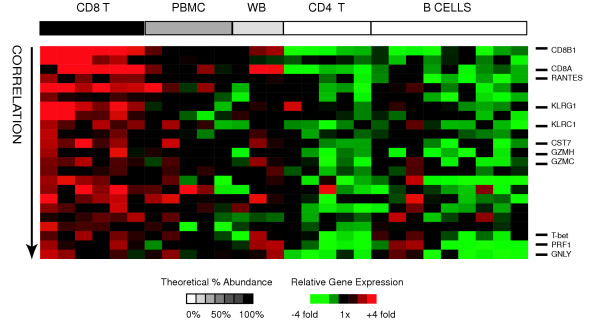
**CD8+ T-cell signature genes**. Relative expression of CD8+ T-cell signature genes is shown for all samples. Genes (rows) are sorted in descending order of correlation of gene expression with relative abundance of T-cells. Samples (columns) are ordered in decreasing relative abundance from left to right. The grayscale bar on top shows the estimated relative abundance of CD8+ T-cells in each class of samples – black indicates 100% relative abundance of CD8+ T-cells, white indicates 0% relative abundance of CD8+ T-cells, and grey indicates intermediate relative abundance of CD8+ T-cells. Gene expression values are centered across all samples to a median of zero. All genes mentioned in the text are listed and their position is marked with a black bar.

CD8+ T-lymphocytes, or cytotoxic T-lymphocytes (CTLs) are involved in cell-mediated cytotoxic reactions, and comprise approximately 35% of T-lymphocytes. We derived a list of CD8+ T-cell specific genes by searching for genes that met the following criteria: I) their expression was significantly positively correlated with relative abundance of CD8+ T-cells across all samples II) their expression was significantly different between CD8+ T-cells and CD4+ T-cells (by SAM) and III) they were at least 2 fold more highly expressed in CD8+ T-cells than in CD4+ T-cells. Using these criteria, we identified 23 "CD8+ signature" genes (represented by 32 clones) (Figure 4). The CD8+ T-cell signature included genes encoding plasma membrane receptors (CD8A/B, IL2RB, KLRC1, KLRG1), cytotoxicity-associated genes (PRF1, GNLY, GZMC/H), and other genes (CCL5/RANTES, T-bet/TBX21, CST7) known to be expressed by CD8+ T-cells [27,28]. The most enriched GO annotations (by EASE analysis) included the cellular component "T-cell receptor complex" and the molecular function "MHC class1 protein binding". CD8+ T-cells appear to be best distinguished from CD4+ T-cells and other cell types based on their cell surface profile and expression of genes encoding proteins with cytolytic functions. While many of the CD8+ T-cell signature genes are also expressed by NK cells [27,29], this signature remains a useful description of the transcriptional activities of resting CD8+ T-cells. The complete list of CD8+ T-cell specific genes and the results of EASE analysis are available as supplemental data (see Additional file 2 for gene list and Additional file 3 for EASE results)

### CD4+ T-cells

The majority of T-lymphocytes are CD4+ "helper" T-cells (the average CD4+/CD8+ T-cell ratio is 1.8) [14], and function primarily in the activation of B-cells and macrophages. We attempted to define CD4+ T-cell specific genes by searching for genes that met the following criteria: I) their expression was significantly positively correlated with relative abundance of CD4+ T-cells across all samples II) their expression was significantly different between CD4+ T-cells and CD8+ T-cells (by SAM) and III) they were at least 2 fold more highly expressed in CD4+ T-cells than in CD8+ T-cells. There were no genes that met all three criteria. Notably, SAM analysis (CD4+ T-cells versus CD8+ T-cells) alone only yielded 4 genes: CD4, ANK3, MXI1, and CTSB. All four genes were more than 2 fold enriched in CD4+ T-cells relative to CD8+ T-cells but only two of these genes (CD4 and ANK3) were also significantly positively correlated with the relative abundance of CD4+ T-cells across all samples. However, since the majority of clones representing CD4 and ANK3 (3 of 4 clones and 2 of 3 clones respectively) did not meet all of the above criteria, these genes did not meet our final criterion of clone consistency (see Methods), and thus did not qualify as CD4+ T-cell "signature" genes (although 2 of the 4 "discordant" CD4 clones did meet the correlation criteria alone). The failure of most CD4 clones to meet our selection criteria may be explained by the fact that CD4 itself is not exclusively expressed by CD4+ T-cells – it is also expressed by monocytes (at lower levels) [30] and by some neutrophils [31]. The one-sidedness of the CD8+ T-cell: CD4+ T-cell comparison suggests that CD8+ and CD4+ T-cells share much of their cellular machinery, and that CD4+ T-cells begin transcription of their effector molecules only upon stimulation while CD8+ T-cells appear to prepare some of their cytotoxic artillery in advance. This result is in contrast to a study of the gene expression profiles of CD4+ and CD8+ T-cells in response to activation, in which as many as 518 genes were found to be preferentially expressed by either CD8+ T-cells or CD4+ T-cells [32].

### Granulocytes

**Figure 5:**
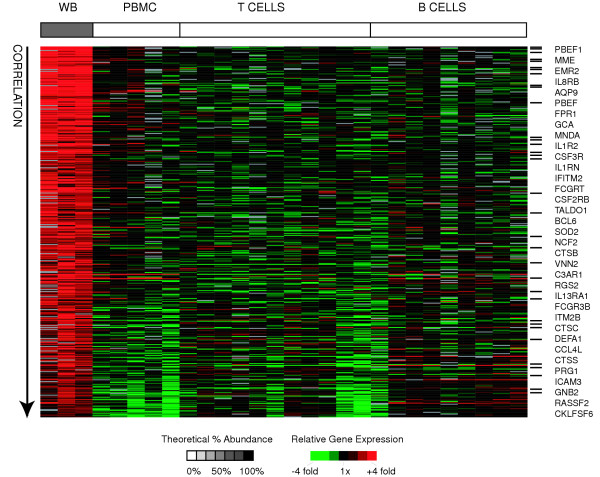
**Granulocyte signature genes**. Relative expression of granulocyte signature genes is shown for all samples. Genes (rows) are sorted in descending order of correlation of gene expression with relative abundance of granulocytes. Samples (columns) are ordered in decreasing relative abundance from left to right. The grayscale bar on top shows the estimated relative abundance of granulocytes in each class of samples – black indicates 100% relative abundance of granulocytes, white indicates 0% relative abundance of granulocytes, and grey indicates intermediate relative abundance of granulocytes. Gene expression values are centered across all samples to a median of zero. All genes mentioned in the text are listed in order of correlation with granulocyte abundance, and black bars indicate their positions in the figure.

Granulocytes, specifically neutrophils, are the most abundant cells in whole blood, but are virtually absent in PBMC samples. They are short-lived, terminally differentiated phagocytic cells involved in innate immunity and acute inflammatory responses. We obtained a granulocyte signature gene list by searching for genes that met the following criteria: I) their expression was significantly positively correlated with relative abundance of granulocytes across all (mixed and purified) samples II) their expression was significantly different between whole blood samples and PBMC samples (by SAM) and III) they were at least 2 fold more highly expressed in whole blood than in PBMCs. We assumed that transcripts more abundant in whole blood than in PBMC samples are most likely to be derived from neutrophils, the most abundant granulocyte, but they could also could also be derived from eosinophils, basophils, platelets, or reticulocytes, because these cell types are also significantly depleted in PBMC relative to whole blood. Using these criteria, we identified 411 putative granulocyte signature genes (represented by 557 clones) (Figure 5). The granulocyte signature contained numerous genes encoding cell surface proteins including: cytokine receptors (CSF2RB, CSF3R, IL1R2, IL1RN, IL8RB, IL13RA1), Fc and complement receptors (FCGRT, FCGR3B/CD16, C3AR1), chemotactic receptors (FPR1, CD10/MME), and EMR2, a recently characterized class B seven-span transmembrane (TM7) receptor, reported to have myeloid specific gene expression [33]. Many of the remaining granulocytes-enriched genes were involved in intracellular pathogen destruction (GCA, PRG1, NCF2, DEFA1, cathepsins B/C/S) or inflammatory mediation (PBEF, MNDA). A number of the genes on this list have been previously shown to be expressed in unstimulated circulating neutrophils (AQP9, BCL6, CD10/MME, CSF2RB, CSF3R, DEFA1, FCGR3B, FPR1, GCA, GNB2, ICAM3, IFITM2, IL8RB, ITM2B, MNDA, NCF2, PBEF, PRG1, RASSF2, RGS2, SOD2, TALDO1, VNN2) [34,35]. The only GO annotations that we identified as statistically enriched with EASE were general biological processes including "inflammatory response" and "innate immune response".

We compared our results to those of a previously published study that also compared whole blood and PBMC samples, and found significant overlap between the two studies. Whitney *et al*. [11] found 704 "granulocyte enriched" genes with mean differences of greater than 2 fold in expression between whole blood and PBMC samples from the same individual. Of the 660 genes in this set that were measured in our study, 171 were also classified as granulocyte signature genes in this study (P < 10^-135^, hypergeometric distribution probability of > = 171 granulocyte genes/660 genes given population ratio of 411 granulocyte genes/16865 genes measured). The concordant genes tended to be those with higher discrimination scores in both studies. The imperfect overlap of the two gene lists is not unexpected because different filtering criteria were used – the 2 fold change required by Whitney *et al*. was one of three criteria in our study. We tested the applicability of our "granulocyte enriched" gene list in an independent data set of 77 whole blood samples from 75 subjects for which the relative abundance of neutrophils was known [11]. For each of the 17046 well-measured clones in this independent data set, we calculated the Pearson correlation of gene expression to neutrophils relative abundance, and converted these correlations to percentile rank correlations. We found that the genes in our granulocyte signature had a median Pearson correlation of 0.17 and a median percentile rank of 0.91 in the other data set, suggesting that the genes in our granulocyte signature are among the most highly correlated with the true neutrophil abundance. The complete granulocyte signature gene list and corresponding EASE analysis results are available as supplemental data (see Additional file 2 for gene list and Additional file 3 for EASE results).

### Lymphocytes

**Figure 6:**
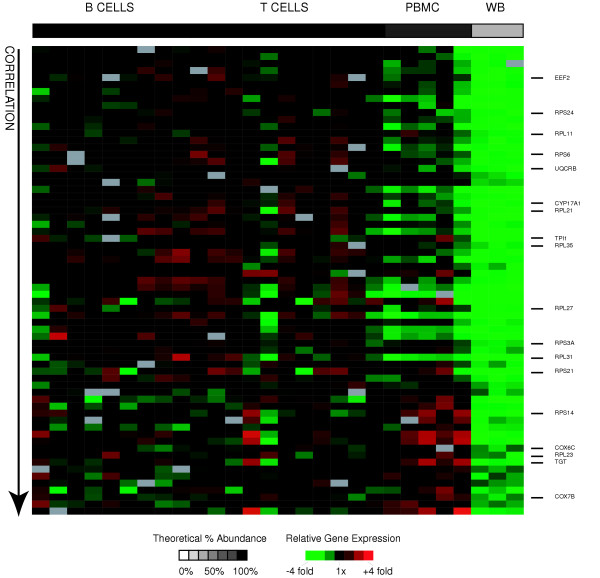
**Lymphocyte signature genes**. Relative expression of lymphocyte signature genes is shown for all samples. Genes (rows) are sorted in ascending order of descending correlation of gene expression with relative abundance of lymphocytes. Samples (columns) are ordered in increasing relative abundance from left to right. The grayscale bar on top shows the estimated relative abundance of lymphocytes in each class of samples – black indicates 100% relative abundance of lymphocytes, white indicates 0% relative abundance of lymphocytes, and grey indicates intermediate relative abundance of lymphocytes. Gene expression values are centered across all samples to a median of zero. All genes mentioned in the text are listed and their position is marked with a black bar.

We derived a lymphocyte signature by searching for genes that met the following criteria: I) their expression was significantly positively correlated with relative abundance of lymphocytes across all samples II) their expression was significantly different in PBMC samples than whole blood samples (by SAM) and III) they were at least 2-fold more highly expressed in PBMC than in whole blood. We assumed that genes more highly expressed in PBMC samples than in whole blood were expressed by lymphocytes, because their relative abundance is approximately 2.5 fold higher in PBMC than in whole blood. Using these criteria, we obtained a lymphocyte signature, consisting of 67 genes (represented by 77 clones) expressed more highly in B-cells, CD4 T-cells, and CD8+ T-cells than in granulocytes (Figure 6). In contrast to the other cell type signature gene lists, this set of genes appeared to contain many universally expressed genes, whose transcripts comprised a particularly large fraction of the total transcripts in these cells, rather than genes known to be specific to a subset of leukocytes. Genes encoding proteins involved in macromolecule biosynthesis, and ribosomal proteins in particular, were strikingly dominant in this signature (CYP17A1, EEF2, RPL11, RPL21, RPL23, RPL27, RPL31, RPL35, RPS14, RPS21, RPS24, RPS3A, RPS6, TGT, TPI1) and were the most statistically significantly over-represented by EASE analysis (top biological process GO annotation was "macromolecule biosynthesis" and top cellular component GO annotation was "cytosolic ribosome"). The dramatic difference in levels of expression of ribosomal proteins between lymphocytes and granulocytes has been previously reported in both SAGE [29,35]and microarray-based studies [11]. Three components of the mitochondrial electron transport chain (UQCRB and COX6C, COX7B) were also more highly expressed in lymphocytes than granulocytes, a finding that is also consistent with a previous microarray study comparing whole blood and PBMC [11]. These findings could reflect either elevated levels of these transcripts in lymphocytes or unusually low levels in granulocytes. Results from a survey of gene expression in 35 human tissue types support the conclusion that lymphocytes have elevated levels of transcripts of genes involved in translation [36]. Perhaps lymphocytes maintain high levels of transcripts encoding the translational apparatus in order to be ready to rapidly ramp up the capacity for protein synthesis when activated. The complete lymphocyte signature gene set and the corresponding EASE analysis results are available as supplemental data (see Additional file 2 for gene list and Additional file 3 for EASE results).

### Individual specific gene expression

Despite the small number of individuals sampled in this study, we carried out a low power search for genes that vary more in expression between individuals than between cell types within an individual. The methods and results of this analysis are available as supplemental data (see Additional file 5 for description, Additional file 6 for text results, and Additional file 7 for figure of results).

## Conclusion

By performing cDNA microarray analysis of purified subpopulations of peripheral blood cells, we were able to obtain global gene expression profiles of peripheral B-cells, T-cells, CD4+ T-cells, CD8+ T-cells, and granulocytes from normal healthy donors. Both unsupervised clustering and supervised statistical analysis yielded sets of genes highly preferentially expressed by each of these cell types. Some characteristics of the genes comprising the signatures we identified for of each of these cell types are summarized in Table 1.

**Table 1:** Gene list summary statistics "Contrasting Cell Pop." is the cell population to which the query cell population was compared in order to define the cell type signature list. "Genes" reports the number of genes that make up the gene expression signature that we defined for each cell type. "Correlation " reports the range and median correlation between expression levels of the signature genes and the relative abundance of the cognate cell type. "Fold Enrichment" reports the range and median ratio between the expression level of the genes in each cell type's signature and the "contrasting cell population" to which it was compared.

**Cell type**	**Contrasting cell pop.**	**Genes**	**Correlation**	**Fold enrichment**
B-cells	T-cells	427	0.95-0.52 (0.81)	16.0-2.0 (2.9)
T-cells	B-cells	222	0.92-0.50 (0.74)	18.4-2.0 (2.8)
CD8+ T-cells	CD4+ T-cells	23	0.91-0.48 (0.67)	16.4-2.0 (2.6)
CD4+ T-cells	CD8+ T-cells	0		
Granulocytes (whole blood)	PBMC	411	0.96-0.37 (0.74)	27.7-2.0 (2.8)
Lymphocytes (PBMC)	Whole Blood	67	0.91-0.49 (0.76)	29.8-2.1 (3.3)

We found that, not surprisingly, the best gene expression based surrogates for the abundance of a particular cell subset were the expression of the genes associated with the characteristic cell surface phenotype (e.g. CD8, CD3D/G, CD20), or other genes known to be associated with cell-type specific functions (such as cytotoxicity or immunoglobulin secretion). While genes previously known to be cell-type specific dominated the cell-type signatures, they also contained numerous unannotated genes, and genes not previously associated with the known functional characteristics of the cells. It is interesting to speculate on the significance of the finding that the lymphocyte subset signatures (B-cells, T-cells, and CD8+ T-cells) were all dominated by genes encoding plasma- membrane associated proteins. This may indicate that, in their resting state, lymphocytes are specialized primarily in the way they sense and monitor their environment. This finding, together with the observation that lymphocytes have elevated levels of transcripts encoding translational machinery, paints a picture of peripheral blood lymphocytes spending the majority of their lives in watchful waiting, while upon activation they respond rapidly are distinguished by their diverse effector roles.

While classical, direct methods of enumerating cell populations remain the simplest, and most accurate method of characterizing the components of mixed cell populations, the cell-type specific gene lists presented here provide an alternative means of characterizing mixed cell populations that could be useful for profiling of archived RNAs and heterogeneous clinical samples for which direct counting is not possible. Furthermore, these cell type signatures contribute significantly to functional annotations of a substantial group of uncharacterized genes. Further studies of the gene expression profiles of other purified leukocyte populations such as NK cells and monocytes will help establish the specialized gene expression programs that enable each cell type to perform its unique function, and will further improve our ability to reconstruct the cellular composition of heterogeneous samples.

## Methods

### Subjects

Blood samples from 3 female and 4 male volunteers were obtained after informed consent. All volunteers were Caucasians, ranging in age from 22–45 years and self reported as free of chronic and acute infections. Complete blood counts were determined at the Stanford University Hospital Clinical Laboratory by automated procedures and have been made available as supplemental data (see Additional file 4). All subjects fell within normal ranges for the major cell populations (% neutrophils, % lymphocytes, % monocytes, % basophils, % eosinophils).

### Isolation and purification of cell types

Purified cell subsets were derived from fresh whole blood of healthy volunteers. We used Ficoll-Paque Plus (Pharmacia Biotech) to enrich for peripheral blood mononuclear cells (PBMC) and incubation with magnetic mAb-coated beads to further select for cell types of interest (MACS, Miltenyi Biotech), according to manufacturer's protocols. When possible, multiple cell types were simultaneously purified from a single donor. CD4+ T-cells and CD8+ T-cells were isolated by negative selection: magnetic depletion of non-target cells, B-cells were prepared both by negative selection (depletion of non-B-cells) and by positive selection – incubation of PBMC with anti-CD19 mAb-coated microbeads. Following purification, viability and was assessed using Trypan Blue and was shown to be >95% in all cases. Purity was assessed using flow cytometry and ranged from ~55–95%, depending on the cell type's abundance in PBMC and the method of selection (positive or negative). The resulting purified cell populations were suspended in a small volume of buffer, flash frozen in liquid N_2 _and stored at -80 C. Each purification yielded at least 1 × 10^6 ^cells of each subtype of interest. Whole blood samples were obtained by extracting RNA from 2.5 ml of with the PaxGene Blood RNA System (Pre-AnalytiX, Hombrechtikon, Switzerland). Total RNA was extracted from frozen PBMC and purified subsets using RNeasy Total RNA Isolation Kit (Qiagen). Table 2 shows the number of subjects and the median purity for each cell subset.

**Table 2:** Sample purity Number of samples and median purity of each purified cell type as a percentage of all cells, measured by FACS. (+) or (-) refer to positive and negative selection respectively.

	**Selection**	**Samples**	**Male**	**Female**	**% Purity**
CD4+ T-CELLS	-	5	2	3	0.91
CD8+ T-CELLS	-	6	2	4	0.86
B-CELLS (-)	-	4	1	3	0.59
B-CELLS (+)	+	5	3	2	0.88
PBMC		5	1	4	N/A
Whole blood		3	2	2	N/A
Overall		28			0.86

### Microarray procedures

Total RNA was linearly amplified twice as previously described [37] with minor modifications. Fluorescent (Cy5-labeled) cDNA probes were prepared from the amplified RNA samples as described [38]and hybridized to cDNA microarrays containing 37,632 array elements, representing approximately 18,000 unique human genes [39]. The cDNA microarrays were manufactured and hybridized as previously described [38,40] (also see [41]). A common reference RNA (Cy3-labeled) was mixed with the Cy5-labeled experimental sample before hybridization to provide a common internal reference standard for comparison of relative gene expression levels across arrays [38,40]. The reference was Stratagene Universal Human Reference RNA – a mixture of RNA from 11 human cell lines (Stratagene, La Jolla, CA). Fluorescence images of the hybridized arrays were obtained using a GenePix 4000B scanner (Axon Instruments, Union City, CA).

### Data extraction and filtering

Gene expression measurements were extracted from the fluorescent array images using GenePix Pro 5.0. Spots with coefficients of variation greater than 0.95 in the Cy3 or the Cy5 channel were excluded from analysis, as were spots that had >15% saturated pixels or that contained fewer than 20 total pixels in either channel. Each array was then subjected to normalization for dye effects: the fluorescence ratios were multiplied by a constant such that the average ratio of Cy5/Cy3 across a subset of high-confidence spots was 1. The criterion for inclusion of data in the initial dataset was a normalized intensity/background ratio of at least 2.5 in the reference channel or the sample channel. Prior to statistical analysis, data analysis was restricted to genes for which at least 75% of the samples had well measured data (as defined by the previously described criteria). This filtering yielded 30,320 clones, corresponding to ~16,865 unique genes (collapsed by gene symbol). For pairwise cell type comparisons with SAM, genes were further required to have 75% good data in each of the relevant cell types. For intrinsic analysis, genes were further required to have well measured data for 2 of the 3 cell types in each of the 5 individuals. For each array, the log ratios were transformed such that the mean log expression ratio of all elements on the array was zero. For all clustering, the measurements for each gene (as log ratios) were centered, by subtracting the mean across all samples, in order to emphasize relative expression within the experimental dataset.

Clones were collapsed to unique genes based on gene symbol whenever possible, and otherwise using IMAGE or LCP (lymphochip) clone numbers. Gene names were converted to gene names and symbols using SOURCE [42]. When reporting "gene" based results for gene lists, the highest scoring (most correlated) clone is reported.

For the signature gene lists and intrinsic gene lists our final filter was the removal of "outlier" genes: genes for which there were 3 or more clones available on the microarray, but only 1 gene was in the results list.

### Significance analysis of microarrays

We used Significance Analysis of Microarrays (SAM) (Version 1) to identify genes whose expression differed significantly between pairs of related cell types [18]. We reported genes that differed between samples at median false discovery rates of 5%. The lymphocyte versus granulocyte and B-cell versus T-cell comparisons employed the two class unpaired analysis, while the CD4 versus CD8 analysis was for five paired samples from the same individual. All comparisons used K-nearest neighbors to impute missing data.

### Pearson correlations

We used a custom perl script to calculate Pearson correlations between the expression levels of each gene and the relative abundance of each cell subset of interest. Since we did not know the true relative abundance of each cell type in each sample, we created relative abundance vectors for each of CD4 T-cells, CD8 T-cells, B-cells, T-cells, and neutrophils based on perfect purifications for the purified populations and average relative abundances for the mixed cell populations. We first calculated the observed Pearson correlation between expression levels for each gene and each abundance vector. We then obtained significance values independently for each gene: parameter pairwise correlation by permuting the query gene/parameter 10,000 times and calculating the percent of permuted correlations that are more extreme (in either direction) than the non-permuted (observed) correlation. We reported genes with p < 0.005 (uncorrected) for the permutation set. Table 3 lists the relative abundance estimates used for correlation calculations.

**Table 3:** Relative abundance estimates used for correlation calculations Estimates of relative abundance of each cell type in each sample were based on perfect purifications for purified cell populations and on normal adult values for mixed populations [14]. We used these estimates in order to identify genes whose measured levels of expression were positively correlated with the estimated relative abundance of each cell type. (Abbrev: WB = whole blood).

	**B-CELLS**	**CD4 T**	**CD8 T**	**WB**	**PBMC**
T-CELLS	0	1	1	0.22	0.55
B-CELLS	1	0	0	0.04	0.10
CD4+ T-CELLS	0	1	0	0.09	0.23
CD8+ T-CELLS	0	0	1	0.13	0.33
LYMPHOCYTES	1	1	1	0.30	0.90
GRANULOCYTES	0	0	0	0.60	0

### EASE GO term annotation

We used EASE [19] to identify themes in the function and cellular localization of genes comprising the signature gene lists for each cell subset. We compared each gene list to the same background file, which consisted of all genes (16,865) that passed the 75% good data filter (described in "Data Filtering" above) for the entire data set. All analyses were done using gene symbol as the unique identifier. In the supplemental data, we report all results for which the Bonferroni corrected Fisher's Exact Probability was less than 0.05 for each of the three ontologies -Molecular Function, Biological Process, and Cellular Component.

## Abbreviations

GO: gene ontology; PBMC: peripheral blood mononuclear cell; SAM: Significance Analysis of Microarrays

## Authors' contributions

CP participated in study design, collected samples, analyzed data, and wrote the manuscript. AAA/MD conceived of the study and participated in study design, sample collection, and manuscript editing. POB participated in experimental design, interpretation of the results, and revision of the manuscript.

## Supplementary Material

Additional File 1 Log_2_(Cy5/Cy3) ratio data for all clones in all experiments. Click here for file

Additional File 2 Complete list of signature genes for each cell population. Click here for file

Additional File 3 Results of EASE analysis for each cell type signature list. Click here for file

Additional File 4 Complete blood counts data for each subject. Click here for file

Additional File 5 Methods and discussion of analysis of individual specific gene expression ("intrinsic" genes). Click here for file

Additional File 6 List of all 410 top "intrinsic" genes. Click here for file

Additional File 7 Figure showing clustering of top 410 "intrinsic" genes. Click here for file
