# Supplementary material for: Cell-type specific gene expression profiles of leukocytes in human peripheral blood
Source: BMC Genomics. 2006 May 16;7:115. doi: 10.1186/1471-2164-7-115 (PMC1479811; doi:10.1186/1471-2164-7-115)
Supplement: Additional File 5 — Methods and discussion of analysis of individual specific gene expression ("intrinsic" genes). [file 1471-2164-7-115-S5.doc]

Supplemental Analysis of Individual Specific Gene Expression

# Overview

We were interested in finding genes that showed consistent expression across different cell types within an individual but differences between individuals (“intrinsic” genes). Such genes may exhibit genetic polymorphisms with gene expression consequences. This analysis was based on a set of five individuals for which we had collected three or more purified cell types from the same individual.

# Methods

We calculated an “intrinsic score” for each of the 30,320 well measured clones on the microarray (quality filters described in Methods: Data Extraction), where the intrinsic score of a given gene was the ratio of the mean squared pair wise difference in that gene's transcript levels between multiple samples from the same individual, to the mean squared pair wise difference in the gene's transcript levels between samples from different individuals. We then averaged the intrinsic score for each unique gene based on gene symbol, and removed genes with fewer than 12 of 15 measurements, producing a set of 16658 intrinsic scores. The analysis was based on five individuals for which 1 CD4, 1 CD8, and 1 or 2 B cell samples were available. For two of the five individuals (females 1 and 2), the B cell samples were obtained on a different day than the T cell samples, and for the remaining subjects, all samples were obtained on the same day. Values were median centered across arrays before variance was calculated. The resulting “intrinsic scores” were normally distributed (p < 10^-15^ Anderson-Darling normality test) with a range of 0.06 to 1.97, a mean of 0.89 and a standard deviation of 0.29. We focused our further analysis on the 164 genes with intrinsic scores that were 2 standard deviations or more below the mean. In order to visualize our results, we clustered the 15 samples and 164 genes according to their expression in the top 164 intrinsic genes.

**Results And Discussion**

Clustering of the samples based on their patterns of expression of the 164 genes with the highest “intrinsic scores” (2 standard deviations from the mean), resulted in grouping of samples by individual rather than by cell type, as shown in Supplemental Figure 1, in contrast to the overview cluster (Manuscript Figure 1). We observed that the two most prominent patterns of “intrinsic” gene expression were gender-associated differences. While this result is not surprising, it is nonetheless noteworthy because it extends our understanding of the cell types in which gender-biased gene expression is exhibited, and gives us confidence in the sensitivity of this method for detecting genes that exhibit the characteristic expression pattern that we are interested in. The majority of the highest scoring genes were Y-linked (CYorf15A, SMCYC, Yorf15B, RPS4Y1, USP9Y), but we also identified numerous genes more highly expressed in the 3 females than the 2 males, with both X-linked and autosomal locations. The X-linked genes included XIST, as well as several other genes that had previously been shown to escape inactivation (ZFX, SYAP1, UTX)[1] as well as several that have not (ALAS2, ARR3). Of the eight named autosomal genes in this set that were more than 2 fold more highly expressed in the 3 females than in males, all but one (LILRB5) had roles in nucleic acid binding (EIF1A, HIST1H1C, HIST1H4C, MRPL23, NAP1L1, SFRS5, ZFP36L2). Another distinct cluster of “intrinsic” genes was a group of genes that is often referred to as “stress response” genes, including JUN, JUNB, CD69, RGS2, and CREM, known for their rapid induction in response various stresses including ex-vivo handling [2]. As discussed in the description of Figure 1, this cluster likely reflects the increased levels of handling to which samples from F3 were subjected and raised the caveat that gene expression patterns that appear to be individual specific expression may in some cases be a result of variations in handling. Most of the genes in the “intrinsic” list belonged to one of the aforementioned clusters, and could thus be explained either by gender differences or by response to handling. A few interesting genes that did not fit either of these patterns were: FRG1 (deleted in facioscapulohumeral muscular dystrophy), DDX17 (involved in RNA metabolism), CBLL1 (ubiquitin protein ligase), ZCCHC2 (putative transcription factor). We did not find any GO annotations to be statistically enriched amongst the entire set of genes with individual specific gene expression (by EASE analysis).

Our results were strikingly different than those of two previous studies of individual specific genes in whole blood samples. The most prominent difference was in genes involved in class II MHC – both studies of whole blood found many of these genes to be individual specific, and were cited as “expected” because of their known sequence polymorphism, yet these genes did not emerge in our analysis [3, 4]. This difference can be explained by the fact that, unlike analysis of whole blood samples, our analysis explicitly required similar expression between B and T cells and thus excluded genes (such as class II MHC) that differ significantly in expression in these two cell types. In fact, our analysis may be considered a more robust selection of genes that exhibit individual specific levels of expression because by using purified cell types, we avoided a major additional source of variation in whole blood studies: variation in relative abundance of cell type composition in whole blood. We did observe, however that the highest scoring “intrinsic” gene in Whitney et al’s whole blood study [3], DDX17, was amongst the highest scoring genes in our study. The list of the top 164 intrinsic genes is available in supplemental data (File S6).

**References**

1. Carrel L, Willard HF: X-inactivation profile reveals extensive variability in X-linked gene expression in females. *Nature* 2005, 434(7031):400-404.

2. Murray JI, Whitfield ML, Trinklein ND, Myers RM, Brown PO, Botstein D: Diverse and Specific Gene Expression Responses to Stresses in Cultured Human Cells. *Mol Biol Cell* 2004, 15(5):2361-2374.

3. Whitney AR, Diehn M, Popper SJ, Alizadeh AA, Boldrick JC, Relman DA, Brown PO: Individuality and variation in gene expression patterns in human blood. *PNAS* 2003, 100(4):1896-1901.

4. Radich JP, Mao M, Stepaniants S, Biery M, Castle J, Ward T, Schimmack G, Kobayashi S, Carleton M, Lampe J *et al*: Individual-specific variation of gene expression in peripheral blood leukocytes. *Genomics* 2004, 83(6):980-988.
